# Highly Drug-Resistant Pathogens Implicated in Burn-Associated Bacteremia in an Iraqi Burn Care Unit

**DOI:** 10.1371/journal.pone.0101017

**Published:** 2014-08-11

**Authors:** Jean-Baptiste Ronat, Jabar Kakol, Marwan N. Khoury, Mathilde Berthelot, Oliver Yun, Vincent Brown, Richard A. Murphy

**Affiliations:** 1 Médecins Sans Frontières/Doctors Without Borders, Paris, France; 2 Department of Health, Sulaymaniyah, Iraq; 3 Ibn Al Haytham Hospital, Amman, Jordan; 4 Albert Einstein College of Medicine, Bronx, New York, United States of America; The Scripps Research Institute and Sorrento Therapeutics, Inc., United States of America

## Abstract

**Objective:**

In low- and middle-income countries, bloodstream infections are an important cause of mortality in patients with burns. Increasingly implicated in burn-associated infections are highly drug-resistant pathogens with limited treatment options. We describe the epidemiology of bloodstream infections in patients with burns in a humanitarian surgery project in Iraq.

**Methods:**

We performed a retrospective, descriptive study of blood culture isolates identified between July 2008 and September 2009 among patients with burns in a single hospital in Iraq who developed sepsis.

**Results:**

In 1169 inpatients admitted to the burn unit during the study period, 212 (18%) had suspected sepsis, and 65 (6%) had confirmed bacteremia. Sepsis was considered the primary cause of death in 198 patients (65%; 95% CI 65–70) of the 304 patients that died. The most commonly isolated organisms were *Pseudomonas aeruginosa* (22 isolates [34%]), *Staphylococcus aureus* (17 [26%]), *Klebsiella pneumoniae* (8 [12%]), *Staphylococcus epidermidis* (7 [11%]), *Acinetobacter baumannii* (6 [9%]), and *Enterobacter cloacae* (5 [8%]). A high proportion of *Enterobacteriaceae* strains produced extended-spectrum beta-lactamase and *S. aureus* isolates were uniformly methicillin-resistant. For gram-negative bacteria, the most reliably active antibiotics were imipenen and amikacin.

**Conclusions:**

Burn patients with sepsis in Iraq were commonly found to have bloodstream pathogens resistant to most antibiotics available locally. Effective empirical therapy of burn sepsis in this region of Iraq would consist of vancomycin or teicoplanin and a carbapenem-class antibiotic with antipseudomonal activity.

## Introduction

Infection remains the leading cause of morbidity and mortality among patients with burns in developing and conflicted-affected countries [1]. The treatment and prevention of infection among burn patients presents a particularly difficult challenge in these contexts. The emergence of highly drug-resistant bacterial pathogens in burn patients represents an alarming new development, with invasive bloodstream infections of particular concern [2]. However, descriptions of the epidemiology of bloodstream infections in burn patients from these regions – data that could potentially improve management strategies – remain scarce.

**Figure 1:**
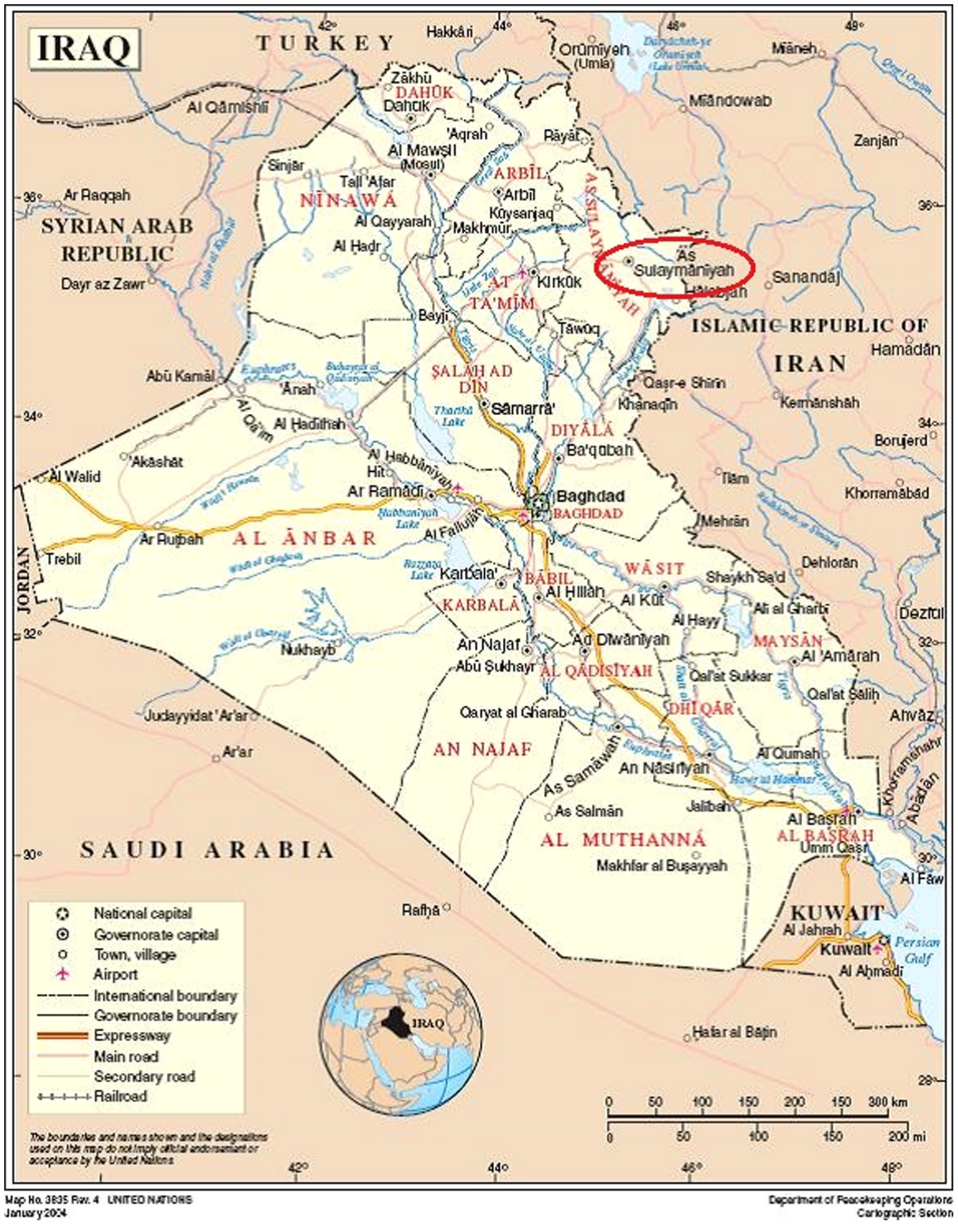
Map of Iraq showing area of Sulaymaniyah.

From 2007 to 2009, Médecins Sans Frontières/Doctors Without Borders (MSF) supported a regional referral burn care center in Sulaymaniyah, part of the Kurdistan region of Iraq (Figure 1). As part of this medical support, an on-site clinical microbiology laboratory was established to improve empirical antibiotics in burn sepsis, to refine clinical decision-making for individual patients, and to improve the understanding of the epidemiology of burn infection in this region.

A study of burns arising from the conflicts in Iraq and Afghanistan described the importance – in burn-associated bacteremia in this region – of *Pseudomonas aeruginosa, Klebsiella pneumoniae, Acinetobacter calcoaceticus-baumannii complex*, and *Staphylococcus aureus*, frequently with multidrug-resistant pattern. In the study, multidrug-resistant organisms were associated with increased mortality, with the largest impact on mortality seen with *Klebsiella pneumonia* [3]. In a recent report from Iran, among patients hospitalized with burn injuries, *Klebsiella pneumoniae* carbapenemase (KPC) was detected in hospitalized burn patients with an associated 33% mortality rate [4].

## Materials and Methods

### Study Setting

In the aftermath of the US military intervention in Iraq, the epicenter of the conflict remained too insecure for the establishment of a medical humanitarian surgical project. As a result, MSF opened several projects outside of the high-conflict zone, including support for a surgical teaching hospital in Sulaymaniyah, with specialization in the treatment of patients with burns. During the period of collaboration from July 2007 to November 2009 between MSF and the Directorate of Health (DOH), the number of patients with burns represented approximately 50–100 admissions per month (2168 admissions).

### Standard Care

All patients arriving to the emergency department were scored for burn severity and area of involvement affected using the Wallace “rule of nines,” and medical resuscitation was initiated. During this period, only patients with burn injuries >10% of total body surface area (TBSA) were admitted. Early eschar excision and grafting was not routinely performed. Initial eschar debridement typically occurred on day 3 or 4, typically followed by delayed skin grafting. Topical antimicrobial therapy consisted primarily of silver sulfadiazine. Systemic antibiotics were not given prophylactically, except perioperatively at which time a single dose was given, but were initiated when burn sepsis or severe local burn infection was suspected. Burn sepsis was defined according to established criteria [5]. Patients with suspected sepsis underwent blood culture collection prior to empirical antibiotic initiation. When burn sepsis was suspected, antibiotics were initiated, using an empirical regimen (vancomycin plus gentamicin or pipericillin/tazobactam plus amikacin) and were adjusted once blood culture results were available approximately 5 days later.

### Study Design

We performed a retrospective descriptive study of burn patients with sepsis syndrome identified at the Sulaymaniyah burn center between July 1, 2008 and September 1, 2009. All burn patients with suspected sepsis who underwent a blood culture series were included. A blood culture series consisted of at least 1 specimen of blood (adults: 10 ml, children's: 2.5–5 ml) obtained from venipuncture for sequential inoculation of bottled media (Aerobic only) in a 24 hour period. Medium bottle stoppers were cleaned with a 70% isopropyl alcohol wipe, which was left in place prior to inoculation [6]. Any repeat sample with the same bacteria on more than one occasion from the same patient was not included in the study. A blood culture was considered to be contaminated if one or more of the following organisms were identified in only one of a series of blood culture specimens: coagulase-negative *Staphylococcus* species, *Propionibacterium acnes*, *Micrococcus* species, viridans group streptococci, *Corynebacterium* species, or *Bacillus* species.

Burn patients from several inpatient departments were included in the study: two intensive care units for adults and children with acute injuries, and two recovery burn wards providing step-down care. Demographic and clinical data were extracted systematically from patient files using standardized instrument.

Blood cultures were processed by a manual standard method (Hemoline performance diphasic, bioMerieux, Marcy l'Etoile, France). Isolates were identified by conventional biochemical methods according to standard microbiological techniques and commercially available kits (API; bioMerieuxVitek, Inc., Hazelwood, MO) [7]. Antibiotic susceptibility was determined by using Kirby Bauer disk diffusion recommended by the Clinical Laboratory Standard Institute [8] and ATB expression, a semi-automatic method (bioMérieux, Marcy l'Etoile, France). Methicillin-resistant *Staphylococcus aureus* (MRSA) and *Enterobacteriaceae* producing extended-spectrum beta-lactamase (ESBL) were confirmed using phenotyping methods [9]. The interpretation of resistance and susceptibility to antimicrobials was determined using CLSI 2008 recommendations [9].

Bacterial etiology and associated drug-resistance profiles were entered into WHONET, a software database tool created by the World Health Organization for antimicrobial resistance surveillance [10]. For external quality control (EQA), half of the isolates were sent to an accredited laboratory in Jordan (Ibn Al Haytham Hospital, Amman, Jordan) for confirmation of bacteria identification and antibiotic susceptibility testing using the MicroScanWalkAway system (Dade Behring, Deerfield, IL).

We defined a multidrug-resistant isolate as any of the following: (1) ESBL-expressing *Enterobacteriaceae* (*Escherichia coli*, Klebsiella spp., Enterobacter cloacae, Proteus spp.); (2) *Pseudomonas aeruginosa* and *Acinetobacter baumannii* isolates non-susceptible to at least one agent in three or more antimicrobial categories typically used for treatment; or (3) MRSA and coagulase-negative *Staphylococcus* (identified in >1 separate specimen). These definitions are consistent with recommendations proposed in 2011 by a panel of international experts [11]. Permission to conduct the study was granted by the regional Department of Health of Sulaymaniyah province.

## Results

Between July 1, 2008 and September 1, 2009, 1169 patients were admitted to the hospital, at a rate of approximately 80–100 patients per month. A total of 795 (68%) patients were female, and the most commonly affected age groups were young adults 15–29 years (31%) and children 0–5 years (31%). Burn characteristics are described in detail below (Table 1).

**Table 1:** Patient demographics and type of injury.

Demographic/Type	N (%)
Gender	1,169
Male	374 (32)
Female	795 (68)
Age	
0–5	362 (31)
6–14	141 (12)
15–29	362 (31)
30–59	257 (22)
≥60	47 (4)
Mechanism of injury	
Fire/flame	421 (36)
Scalding	631 (54)
Contact	82 (7)
Other	35 (3)
Place of injury	
Home (including yard)	970 (83)
Work	129 (11)
School/outdoors	70 (6)
Alleged intent of injury	
Accidental, self	946 (81)
Accidental, other	129 (11)
Intentional, self	82 (7)
Intentional, other	12 (1)
% TBSA burned (median 18%, IQR 9.5–39.0)	
≤15	338 (29)
15–30	468 (40)
31–50	234 (20)
51–69	82 (7)
≥70	47 (4)
Hours between injury and presentation (median, IQR)	1, (0.5–1.5)

TBSA, total body surface area.

We analyzed 389 blood samples among 212 patients with suspected sepsis. Among these 212 patients, 65 (31%) had a proven bloodstream infection with the causative agent cultured from blood samples, while 112 (53%) patients had negative blood cultures. Thirty-five (16%) patients had blood samples that were consistent with our definition of contamination. The 65 patients with proven bacteremia represented 6% of the 1169 total inpatients treated in the burn center during the study period. Median number of positive blood cultures per person in those with any positive blood cultures was 1.5 (IQR, 1–2); no case of polymicrobial bacteremia were observed. Forty-four (68%) of the 65 patients with positive blood cultures were in the adult burn unit, 10 (15%) patients in the pediatric unit, and 11 (17%) in recovery units (step-down care). Bloodstream infection rates rose by TBSA involved in burn (Table 2). Proven bacteremia was observed in 11 (18%) patients with 15–30% TBSA involved, 20 (30%) with 31–50% TBSA involved and 34 (52%) with 51–69% TBSA involved.

**Table 2:** Patient care outcomes and mortality.

Outcome	N (%)
	N = 1169
Length of hospital stay: median 8 days, IQR 3–14	
Admission outcome	
Recovery	772 (66)
Death	303 (26)
Discharged against medical advice	94 (8)
Cause of death	
Inhalation injury	106 (35)
Sepsis	198 (65)
Mortality by % TBSA burned	
≤15	0 (n/a)
15–30	70 (15% died)
31–50	119 (51% died)
51–69	78 (95% died)
≥70	47 (100% died)
Proven bacteremia (n = 65) by % TBSA burned (median 59%, IQR 36.5–77.0)	
≤15	0 (n/a)
15–30	11 (18% sepsis)
31–50	20 (30% sepsis)
51–69	34 (52% sepsis)
≥70	0 (n/a)

TBSA, total body surface area.

The median hospital admission duration was 8 days (IQR, 3–14). An overall mortality rate of 26% (95% CI, 24–29%) was observed (Table 2). Mortality was considered by TBSA involved: 0 (0%) among patients presented with <15% BSA involvement, 70 patients (15%) with 15–30% TBSA, 119 patients (51%) with 31–51% TBSA, 125 patients (97%) with >51% BSA.

Gram-negative organisms represented 41 (63%) of 65 overall isolates (Table 3). The most common gram-negative organisms were *Pseudomonas aeruginosa* (34%), *Klebsiella pneumoniae* (12%), *Acinetobacter baumannii* (9%), and *Enterobacter cloacae* (8%). Gram-positive bacteria represented 24 (37%) of 65 of overall isolates. The most common gram-positive organisms were *Staphylococcus aureus* (26%) and *Staphylococcus epidermidis* (11%). Forty-eight (74%) of 65 patients were found to have a multidrug-resistant organism including 63% of the gram-negative isolates (Table 3). ESBL-expression was observed in 8 of 13 *Enterobacteriaceae* isolates.

**Table 3:** General bacteria etiology among blood culture isolates.

Organism	N = 65	
	n	%
Gram-negative bacteria	41	63
Gram-positive bacteria	24	37
Specific organisms identified		
*Pseudomonas aeruginosa*	22	33
*Staphylococcus aureus*	17	26
*Klebsiella pneumoniae*	8	12
*Acinetobacter baumannii*	9	14
*Staphylococcus epidermidis*	7	11
*Enterobacter cloacae*	5	8

Among gram-negative bacteria, the most reliably active drugs were imipenem and amikacin which were active against 83% and 61% of isolates, respectively (Table 4). Overall 92% of gram-positive isolates were multidrug-resistant (Table 5) reflecting the prominent role of MRSA bloodstream infections in this patient population. Among *S. aureus*, three more reliably active drugs were vancomycin, rifampicin, and clindamycin, which were active against 100%, 71%, and 65% of isolates, respectively (Table 6). A high level of concordance was noted among 47 samples that were sent to the reference laboratory (Unweighted Cohen Kappa 0.92, IC 95 [0.90–0.94]).

**Table 4:** Resistance profile for gram-negative bacteria.

Organisms	*K.pneumoniae*	*E. cloacae*	*P. aeruginosa*	*A. baumannii*
	N = 8	N = 5	N = 22	N = 6
	n (%R)	n (%R)	n (%R)	n (%R)
Ampicillin/Sulbactam				1 (17)
Amoxicillin/Clavulanic ac	4 (50)			
Ticarcillin/Clavulanic ac	8 (100)	5 (100)	16 (73)	5 (83)
Piperacillin		5 (100)	14 (64)	6 (100)
Piperacillin/Tazobactam	7 (87)	2 (40)	9 (41)	4 (67)
Cephalothin (CIG)	8 (100)			
Cefuroxime (CIIG)	8 (100)	5 (100)		
Ceftazidime (CIIIG)	7 (87)	5 (100)	12 (54)	4 (67)
Cefepime (CIVG)	4 (50)	4 (80)	7 (32)	3 (50)
Gentamicin	8 (100)	4 (80)	21 (95)	6 (100)
Amikacin	6 (75)	1 (20)	5 (23)	4 (67)
Ciprofloxacin	5 (62)	1 (20)	14 (64)	3 (50)
Co-trimoxazole	4 (50)	4 (80)		
Imipenem	0 (0)	0 (0)	5 (23)	2 (33)
Colistin			0 (0)	0 (0)

**Table 5:** Proportion of blood culture isolates that were multidrug-resistant.

Organism	Multidrug-resistant isolates	
	n/N	%
Gram-negative bacteria	26/41	63
Gram-positive bacteria	22/24	92
Specific organisms identified		
*Pseudomonas aeruginosa*	14/22	64
*Staphylococcus aureus*	17/17	100
*Klebsiella pneumoniae*	4/8	50
*Acinetobacter baumannii*	4/9	67
*Staphylococcus epidermidis*	5/7	71
*Enterobacter cloacae*	4/5	80
**Total multidrug-resistant organisms**	**48/65**	**74**

**Table 6:** Resistance profile of Gram-positive bacteria.

Organism	*S. aureus*	*S. epidermidis*
	N = 17	N = 7
	n (%R)	n (%R)
Penicillin G	17 (100)	7 (100)
Oxacillin	17 (100)	5 (71)
Gentamicin	15 (88)	2 (29)
Fusidic acid	12 (71)	3 (43)
Levofloxacin	10 (59)	1 (14)
Clindamycin	6 (35)	0 (0)
Minocycline	5 (29)	1 (14)
Rifampin	5 (29)	0 (0)
Quinupristin/Dalfopristin	2 (12)	0 (0)
Nitrofurantoin	0 (0)	0 (0)
Vancomycin	0 (0)	0 (0)

## Discussion

We report a high prevalence of multidrug-resistant strains notably *Pseudomonas aeruginosa*, ESBL-expressing *Enterobacteriaceae*, and MRSA among patients with burn-associated bacteremia in northern Iraq. Our report confirms and strengthens prior data describing causes of burn-related bacteremia in this region [12]. The high prevalence of multidrug-resistant organisms gives serious cause for concern particularly because most isolates are resistant to the antimicrobial agents generally used in the region for empirical treatment of burn sepsis. Importantly, the use of blood culture samples illustrates that multidrug-resistant organisms are not merely skin or wound colonizers but in this region are the dominant cause of invasive bloodstream infections in burn patients.

In contrast to resource-rich settings, where *S. aureus* is the most common cause of burn-associated bacteremia and sepsis, gram-negative bacteremia was shown to be common in this region of Iraq [13], [14]. Gram-negative infections are frequently lethal in burn patients in resource-limited settings [15], particularly where early excision and grafting is not routinely practiced and access to appropriate intravenous antimicrobials is lacking.

Based on these findings from burn patients with suspected sepsis in this region, where basic hygiene, isolation, and early excision may be lacking, the use of empirical therapy combining a carbapenem and an aminoglycoside such as amikacin may be warranted. The use of this regimen could potentially be supported by a mortality study performed in the same burn unit in 2007, which described a mortality rate of 20% of patients with burn-associated sepsis [16].

This study has several strengths. The use of blood cultures helped to show the presence of multidrug-resistant organisms in invasive burn infections. The microbiology laboratory adhered to international standards, and a reference laboratory was employed for quality control. A high level of concordance was noted for samples sent to the reference laboratory. The study also has several weaknesses. We were unable to correlate blood isolates with severity and patient outcomes owing to a lack of data linking isolates with patient characteristics such as burn TBSA area involvement and mortality. The study was retrospective, and cultures were obtained at the discretion of the attending surgeons, which allowed for the possibility of sampling bias. Such a bias could have resulted in the inclusion of more severely ill patients and exclusion of less severely ill patients.

The introduction of clinical microbiology capacity in this northern Iraq burn hospital was advantageous in several ways. In patients with less-resistant pathogens, the introduction of microbiology laboratory capacity allowed for the simplification of therapy once the specific pathogen was isolated. Moreover, the aggregated culture data, with the use of WHONET software, allowed physicians to improve the choice of initial antibiotics in patients with suspected burn sepsis pending microbiology results. The culture data helped the hospital order drugs in a more rational way. Although routine microbiology is an expensive undertaking for humanitarian surgical projects, these data suggest there is added value in acquiring microbiology data to improve treatment strategies and the understanding of antibiotic resistance in poor and conflict-affected settings.

Infection control must be prioritized in burn units in Iraq to potentially halt or reverse the rapid evolution of antibiotic resistance. Selection and cross-transmission of antibiotic-resistant pathogens occurs readily in inpatient burn units in the developing world. Basic hygiene standards are often not observed uniformly in inpatient burn units in resource-limited settings, creating conditions favoring the cross-transmission of drug-resistant bacteria. More research is needed on strategies to reduce colonization and infection with multidrug-resistant organisms in contexts where early excision and grafting are not routine, space constraints are great, and access to disposable materials is limited.

We found that multidrug-resistant organisms were common invasive pathogens in burn-injury patients with bacteremia in northern Iraq. Burn treatment programs in Iraq and similar regions, whether operated by nongovernmental organizations or by the government, must respond with appropriate use of antimicrobials and invest additional resources in hospital infection control and surveillance of antibiotic resistance. Our results demonstrated that on-site microbiology, although costly, provided critical data for refining individual patient care through more rational use of antimicrobials and improved empirical treatment of burn-associated sepsis in this region.
